# Bibliometric analysis of research hotspots and emerging trends in microRNAs and atherosclerosis (2007–2025)

**DOI:** 10.3389/fcvm.2026.1770481

**Published:** 2026-03-17

**Authors:** Jiuhua She, Yuxue Yu, Boyan Gao, Jiayuan Song, Liyuan Cao, Meiling Wang, Xiaoli Yan, Liping Chang, Guijun Shi

**Affiliations:** 1 College of Chinese Medicine, Changchun University of Chinese Medicine, Changchun, China; 2 College of Integrative Medicine, Changchun University of Chinese Medicine, Changchun, China; 3 Department of Endocrinology, Changchun Chinese Medicine Hospital, Changchun, China; 4 Department of Integrative Medicine, Ji Lin Cancer Hospital, Changchun, China; 5 Department of Cardiovascular Medicine, Affiliated Hospital of Changchun University of Chinese Medicine, Changchun, China

**Keywords:** atherosclerosis, bibliometrics, cellular behavior, immune regulation, inflammation, lipid metabolism, microRNAs

## Abstract

**Background:**

An increasing number of studies have demonstrated that microRNAs (miRNAs) play critical roles in atherosclerosis (AS) and have emerged as promising therapeutic targets in this disease. Through a bibliometric approach, we constructed a comprehensive analytical framework to systematically explore key research topics and emerging trends in this field.

**Methods:**

Relevant literature was first retrieved from three core databases, namely the Web of Science Core Collection (WOSCC), Scopus, and PubMed. Duplicate records among the retrieved literature were removed using Python (version 3.11). Subsequent data analysis was performed using RStudio, CiteSpace, VOSviewer, WPS Office, and SciMAgo Graphica. The specific analytical dimensions included the following: co-occurrence analysis to determine the frequency of key elements such as countries, regions, and institutions; keyword clustering analysis; and burst detection analysis to identify research trends and hotspots in this field.

**Results:**

A total of 3,478 publications published between 2007 and 2025 were included in the analysis. China and the United States were identified as the most influential countries, contributing the highest number of publications in this field. Christian Weber and Carlos Fernández-Hernando were recognized as leading experts based on their high publication output. Keyword analysis revealed that miRNAs are primarily involved in regulating AS-related processes, including inflammation and immune responses, lipid metabolism, and cellular functions. Future research is expected to increasingly focus on the translational applications of miRNAs in AS. The expression levels of specific circulating miRNAs could serve as biomarkers for the diagnosis of AS or the prediction of disease prognosis. In clinical applications, greater emphasis is expected to be placed on the development of novel materials for the targeted delivery of miRNA-based therapeutics to AS lesions.

**Discussion:**

This study employed bibliometric methods to analyze the applications of miRNAs in AS, elucidate research trends and frontiers, and provide guidance for the optimization of therapeutic strategies.

## 1 Introduction

Cardiovascular diseases (CVDs) remain the leading cause of mortality and morbidity worldwide, imposing a substantial burden on global healthcare systems and society (1, 2). AS, the fundamental pathological basis underlying CVDs, is characterized by lipid accumulation, chronic inflammation, and fibrous plaque formation (3). The pathogenesis of AS involves a complex and chronic inflammatory cascade characterized by endothelial dysfunction, lipid accumulation, inflammatory activation, vascular smooth muscle cell (VSMC) proliferation, and fibrosis (4–7). Therefore, comprehensive therapeutic strategies targeting multiple molecular and cellular pathways are essential for the effective management of AS.

MiRNAs, a class of endogenous non-coding RNAs, have emerged as pivotal regulators of disease pathogenesis and diagnostic development by modulating gene expression through mRNA targeting (8). Since the discovery of lin-4 in 1993, a total of 1,917 human miRNAs have been identified to date (9, 10). These miRNAs participate extensively in lipid metabolism, inflammation, and other pathophysiological processes, thereby playing crucial roles in the regulation of CVD-related molecular mechanisms. For instance, miR-33 can target and inhibit the expression of ATP-binding cassette transporter A1 (ABCA1) and ATP-binding cassette transporter G1 (ABCG1), thereby impairing reverse cholesterol transport and promoting foam cell formation and plaque progression (11, 12). Endothelial miR-126-5p maintains the proliferative reserve of endothelial cells (ECs) by targeting delta-like 1 homolog (Dlk1), thereby suppressing atherosclerotic lesion formation (13). Furthermore, recent evidence has uncovered a novel protective role of miR-126-5p in ECs. MiR-126-5p forms a ternary complex with Argonaute-2 (Ago2) and Mex-3 RNA binding family member A (Mex3a), which is transported into the nucleus via autophagic vesicles. In the nucleus, miR-126-5p dissociates from Ago2 and directly binds to caspase-3, thereby inhibiting its activation. This process reduces endothelial apoptosis and exerts protective effects against atherosclerosis (14, 15). Further studies have demonstrated that miR-204-5p can target and inhibit the expression of scavenger receptor A (SR-A), while miR-204-3p suppresses CD36 transcription in the nucleus; these two subtypes synergistically block lipid uptake by macrophages, suggesting that miR-204 inhibits foam cell formation and disease progression (16, 17).

Bibliometrics is a quantitative analytical method that enables a comprehensive understanding of a scientific field through multilevel evaluation and visualization techniques (18). However, bibliometric analyses focusing on the applications of miRNAs in AS remain limited. Therefore, we conducted a bibliometric analysis based on CiteSpace, VOSviewer, and Scimago Graphica (19–21). Visualization techniques were employed to map collaborative networks in miRNA research on AS across countries, regions, institutions, authors, and journals; to summarize the current research landscape; to identify research hotspots and emerging trends; and to provide insights into future research directions.

## 2 Methods

### 2.1 Sources of research data and retrieval scheme

The selection of appropriate academic databases and the formulation of an effective retrieval strategy are critical for obtaining high-quality datasets. In this study, three authoritative databases—WOSCC, Scopus, and PubMed—were employed to provide a comprehensive dataset for bibliometric analysis. WOSCC, which encompasses Science Citation Index Expanded (SCIE), Social Sciences Citation Index (SSCI), Arts & Humanities Citation Index (A&HCI), and Emerging Sources Citation Index (ESCI), offers a wealth of authoritative publications related to miRNAs and AS. Additionally, Scopus is widely recognized as the largest abstract and citation database of peer-reviewed literature, covering a broad spectrum of academic disciplines. As a specialized repository, PubMed provides an extensive and continuously expanding archive of journal literature in the biomedical and life sciences fields. Therefore, a systematic search across the three databases helps comprehensively present the overall developmental landscape of miRNA research in AS.

**Figure 1:**
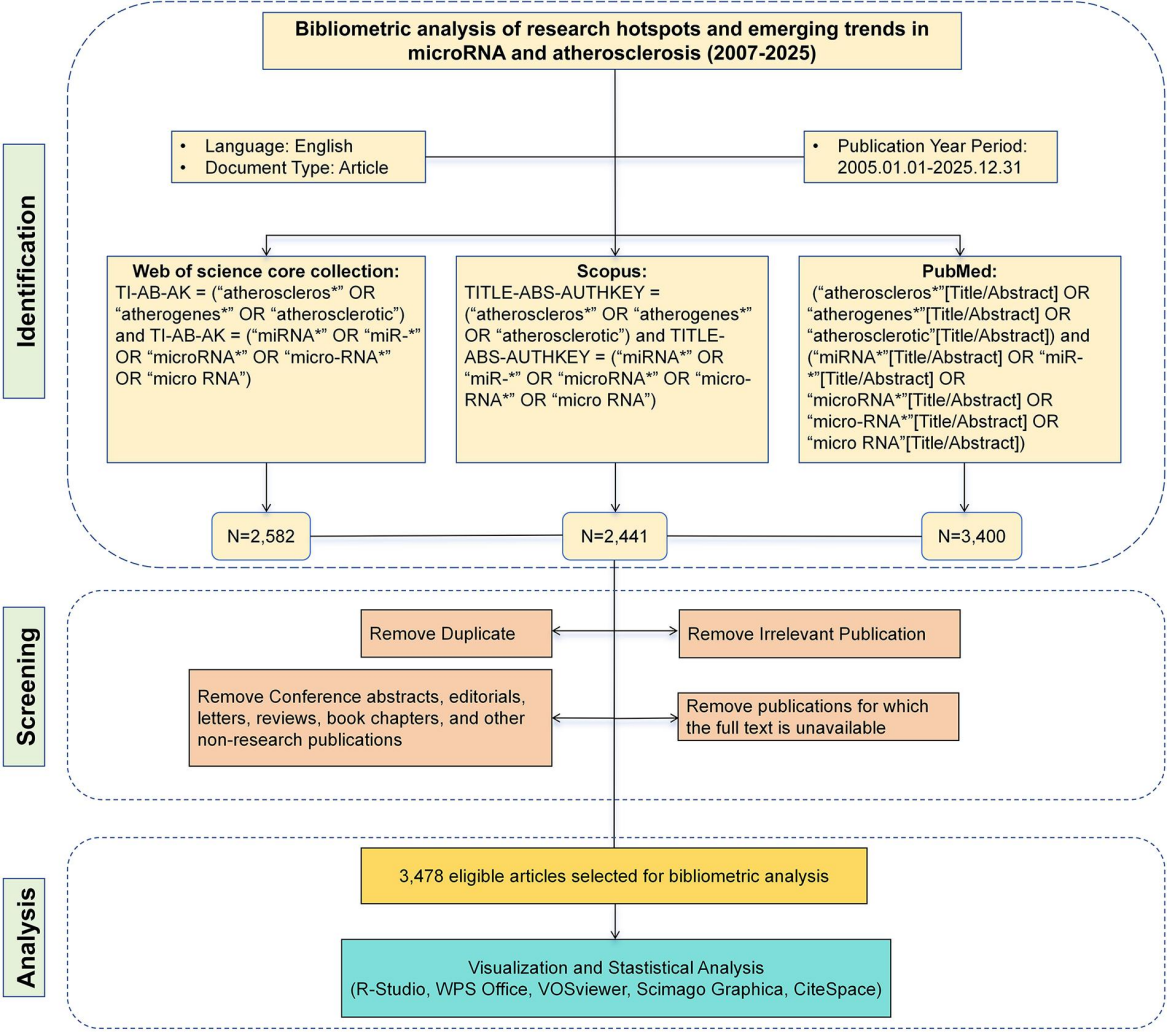
Flowchart of literature search, screening, and data analysis.

wBased on the selected databases, this study developed independent yet methodologically consistent search strategies for each database. The search focused on the two core concepts of “atherosclerosis” and “miRNA.” To cover as many relevant term variations as possible, search terms were combined using wildcards and Boolean operators. To enhance thematic relevance, the search scope was uniformly restricted to the title, abstract, and author keyword fields. Automatically generated index terms (e.g., Keywords Plus in WOSCC) were excluded to avoid potential bias from non-manual indexing. Specifically, the search in WOSCC was limited to the TI, AB, and AK fields; Scopus covered TITLE, ABS, and AUTHKEY; and PubMed was restricted to the Title/Abstract field. Only English-language articles were included. All searches were conducted on January 14, 2026, and covered publications from January 1, 2005, to December 31, 2025. Table 1 details the specific search strategy for each database, and Figure 1 illustrates the overall literature screening process.

**Table 1:** Search strategies for each database.

Databases	Search strategies
Web of science core collection	#1 TI-AB-AK = (“atheroscleros*” OR “atherogenes*” OR “atherosclerotic”)
	#2 TI-AB-AK = (“miRNA*” OR “miR-*” OR “microRNA*” OR “micro-RNA*” OR “micro RNA”)
	#3 Document Type=Article
	#4 Language=English
	#5 PY = (2005–2025)
	#6 #1 AND #2 AND #3 AND #4 AND #5
Scopus	#1 TITLE-ABS-AUTHKEY=(“atheroscleros*” OR “atherogenes*” OR “atherosclerotic”)
	#2 TITLE-ABS-AUTHKEY=(“miRNA*” OR “miR-*” OR “microRNA*” OR “micro-RNA*” OR “micro RNA”)
	#3 Document Type=Article
	#4 Language=English
	#5 PY = (2005–2025)
	#6 #1 AND #2 AND #3 AND #4 AND #5
PubMed	#1 (“atheroscleros*”[Title/Abstract] OR “atherogenes*”[Title/Abstract] OR “atherosclerotic”[Title/Abstract])
	#2 (“miRNA*”[Title/Abstract] OR “miR-*”[Title/Abstract] OR “microRNA*”[Title/Abstract] OR “micro-RNA*”[Title/Abstract] OR “micro RNA”[Title/Abstract])
	#3 Document Type=“Adaptive Clinical Trial” or “Clinical Trial” or “Clinical Trial, Phase I - Ⅳ” or “Clinical Trial, Veterinary” or “Controlled Clinical Trial” or “Equivalence Trial” or “Multicenter Study” or “Pragmatic Clinical Trial” or “Randomized Controlled Trial” or “Randomized Controlled Trial, Veterinary” or “Case Reports” or “Clinical Study” or “Comparative Study” or “Evaluation Study” or “Observational Study” or “Observational Study, Veterinary” or “Twin Study” or “Validation Study” or “Clinical Trial Protocol” or “Dataset” or “Preprint” or “Technical Report”
	#4 Language=English
	#5 PY = (2005–2025)
	#6 #1 AND #2 AND #3 AND #4 AND #5

### 2.2 Data processing

A total of 8,423 publications were initially retrieved, including 2,582 from WOSCC, 2,441 from Scopus, and 3,400 from PubMed. Two independent reviewers (YYX and GBY) conducted a thematic relevance screening of all publications. Any discrepancies were resolved by a third reviewer (SJY).

Inclusion Criteria: (1) Study type: original research articles. (2) Research subject/topic: studies must cover both of the following core aspects: (a) miRNA-related research; (b) AS research involving human trials, animal models, or cellular mechanistic experiments. (3) Language: English. (4) Publication period: January 1, 2005, to December 31, 2025.

Exclusion Criteria: (1) Publication types: conference abstracts, editorials, letters, reviews, book chapters, and other non-original research publications were excluded. (2) Publications not relevant to the research topic. (3) Publications with unavailable full text.

After excluding irrelevant studies, bibliographic data of the remaining publications were downloaded in plain text and CSV formats. The dataset included comprehensive metadata such as title, author, affiliation, country, publication year, abstract, keywords, and references. To harmonize data structures across databases, Python (version 3.11) was used to convert Scopus CSV files into a plain text format consistent with WOSCC and PubMed, ensuring uniformity in record presentation and reference formatting. Data cleaning was conducted using Python (version 3.11), including: deduplication based on DOIs; removal of records with “[Anonymous]” in the author field; exclusion of entries affiliated with non-research entities [e.g., the Egyptian Knowledge Bank (EKB)]; standardization and merging of duplicate affiliation names; and elimination of retracted publications. Ultimately, 3,478 publications were included for subsequent bibliometric analysis.

### 2.3 Data analysis

Table 2 summarizes the bibliometric tools used in this study and their corresponding analytical purposes for miRNA research in AS. R-Studio (Version 4.5.2) was used to analyze basic bibliometric characteristics, publication journals, and references. WPS Office (Version 12.1.0.24657) was used to analyze and visualize annual publication trends. VOSviewer (Version R1.6.20) was applied to analyze countries/regions, institutions, authors, cited authors, cited journals, and keywords. Scimago Graphica (Version 1.0.53) was used to analyze scientific output and collaboration patterns of countries/regions. CiteSpace (Version 6.3.R1) was used for visualizing inter-institutional collaboration, author co-occurrence, journal dual-map overlay, cited literature network co-occurrence, cited literature cluster analysis, and keyword co-occurrence, clustering, timeline, and burst detection.

**Table 2:** Bibliometric tools and their analytical purposes in this study.

Bibliometric Tool	Version	Application in This Study
RStudio	4.5.2	analyze the basic bibliometric characteristics
		analyze the publication journals
		analyze the references in the research field
WPS Office	12.1.0.24657	analyze and visualize the annual publication trends
VOSviewer	R1.6.20	analyze countries and regions
		analyze institutions
		analyze authors
		analyze cited authors
		analyze cited journals
		analyze keywords
Scimago Graphica	1.0.53	visualization of scientific output and collaboration patterns of countries and regions
CiteSpace	6.3.R1	visualization of inter-institutional collaboration visualization of author co-occurrence visualization of journal dual-map overlay visualization of cited literature network co-occurrence and cited literature cluster analysis visualization of keyword co-occurrence, clustering, timeline and burst detection

## 3 Results

### 3.1 Bibliometric characteristics and annual publication growth trends

**Figure 2:**
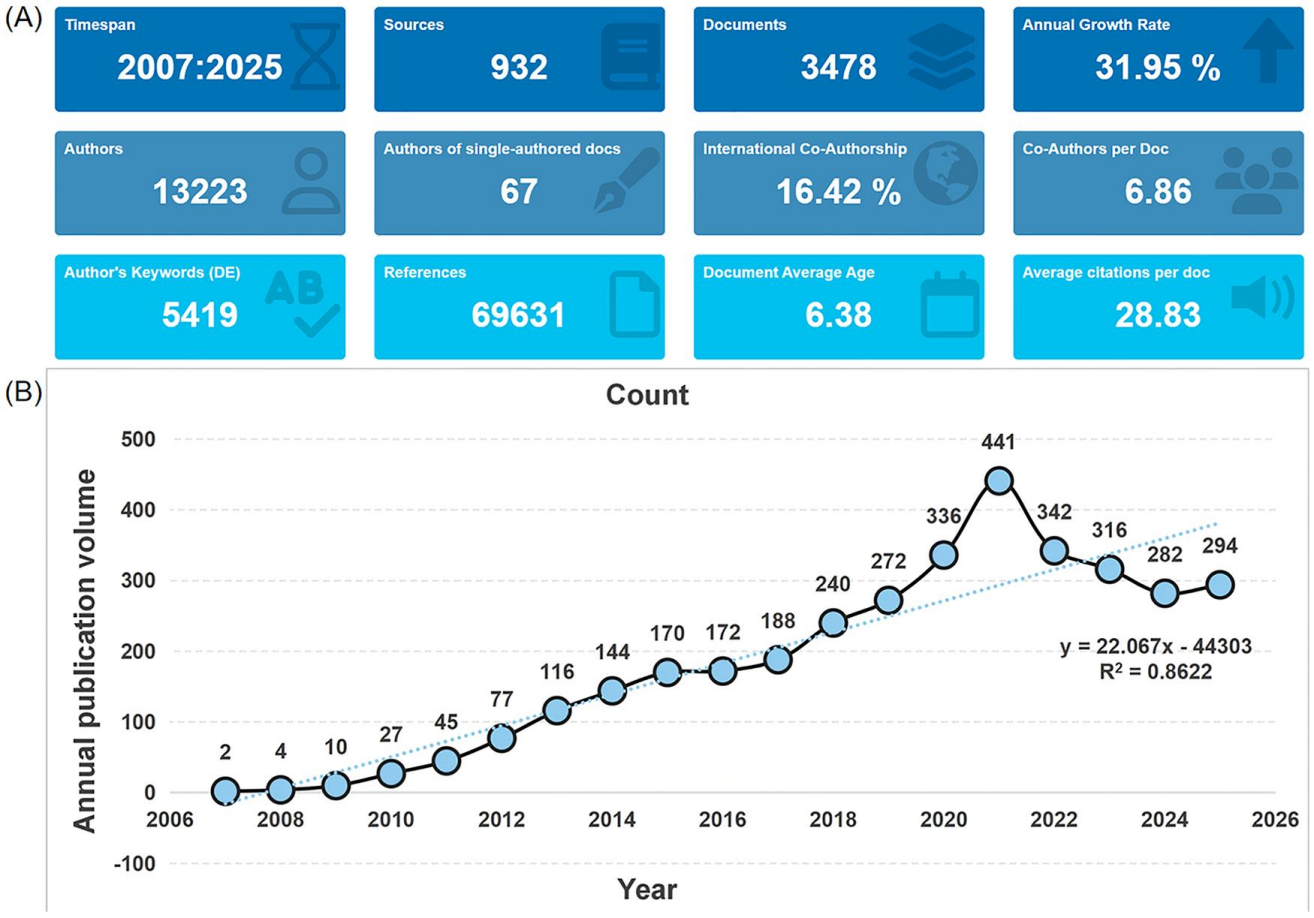
Bibliometric summary and annual publication trends in microRNA and atherosclerosis research. **(A)** Core bibliometric indicators, including the number of publications, authors, journals, and other related metrics. **(B)** Annual publication volume from 2007 to 2025, with a linear regression line showing the overall growth trajectory.

Figure 2A presents a bibliometric overview of the research field over the 18-year period from 2007 to 2025. The results indicate that the field has shown a steady growth trend, with a total of 3,478 publications from 932 journals, suggesting that the field has formed a certain research accumulation and maintained continuous development. The annual growth rate of publications was 31.95%, indicating a gradual increase in attention from the global academic community to this research area. Author collaboration analysis showed that a total of 13,223 authors participated in relevant research, among which 67 were single-authored papers, demonstrating that team collaboration is the main research model in this field. Each document had an average of 6.86 co-authors, with an international co-authorship rate of 16.42%, reflecting two collaboration modes in the field: intra-institutional and cross-border cooperation. In terms of knowledge accumulation, 5,419 author keywords and 69,631 cited references were extracted from the field, reflecting the diversity of research topics and the knowledge accumulation level of the field. Academic impact analysis showed that the average number of citations per included document was 28.83, with a mean publication age of 6.38 years, indicating that the research outputs in this field have sustained scholarly relevance and stable citation activity.

Figure 2B illustrates the yearly distribution of publications in this field. A total of 3,478 publications were published between 2007 and 2025. Starting with only 2 publications in 2007, the number steadily increased, peaking at 441 publications in 2021. The linear regression model (y = 22.067×−44,303, R^2^ = 0.8622) indicated a strong positive correlation between publication volume and time, with an average annual growth of approximately 22 publications. In recent years, annual output has remained relatively stable at a high level. Owing to its distinctive research value and promising development potential, this field continues to attract growing scholarly attention, thereby fostering novel research directions and theoretical advancements.

### 3.2 National and regional distribution and institutional collaboration networks

Globally, 84 countries and regions have contributed to research in this field, collectively publishing 3,478 papers. The geographical distribution of research output is summarized in Table 3, highlighting the top 15 countries/regions, each contributing over 40 publications. China and the United States emerged as the leading contributors, exhibiting high research productivity. Specifically, China published 1,903 papers, followed by the United States with 599, ranking first and second globally in total output. The dominance of China and the United States in research output may be largely attributable to the high prevalence of AS in these populations. As the principal pathological basis of life-threatening CVDs, such as myocardial infarction and stroke, AS represents a major public health burden in both countries. Escalating direct medical expenditures associated with AS further exacerbate the economic burden in these countries.

**Table 3:** Top 15 countries and regions in terms of number of publications.

Rank	Documents	Citations	Year	Country/regions
1	1,903	47,064	2009	China
2	599	34,054	2007	USA
3	167	11,683	2008	Germany
4	156	4,073	2008	Italy
5	108	3,201	2012	Spain
6	99	803	2015	Iran
7	93	6,487	2009	United Kingdom
8	92	4,327	2010	Canada
9	69	684	2014	India
10	64	2,334	2009	Japan
11	64	327	2012	Poland
12	53	5,806	2009	Netherlands
13	50	1,777	2007	South Korea
14	42	1,378	2011	France
15	42	354	2015	Russia

**Figure 3:**
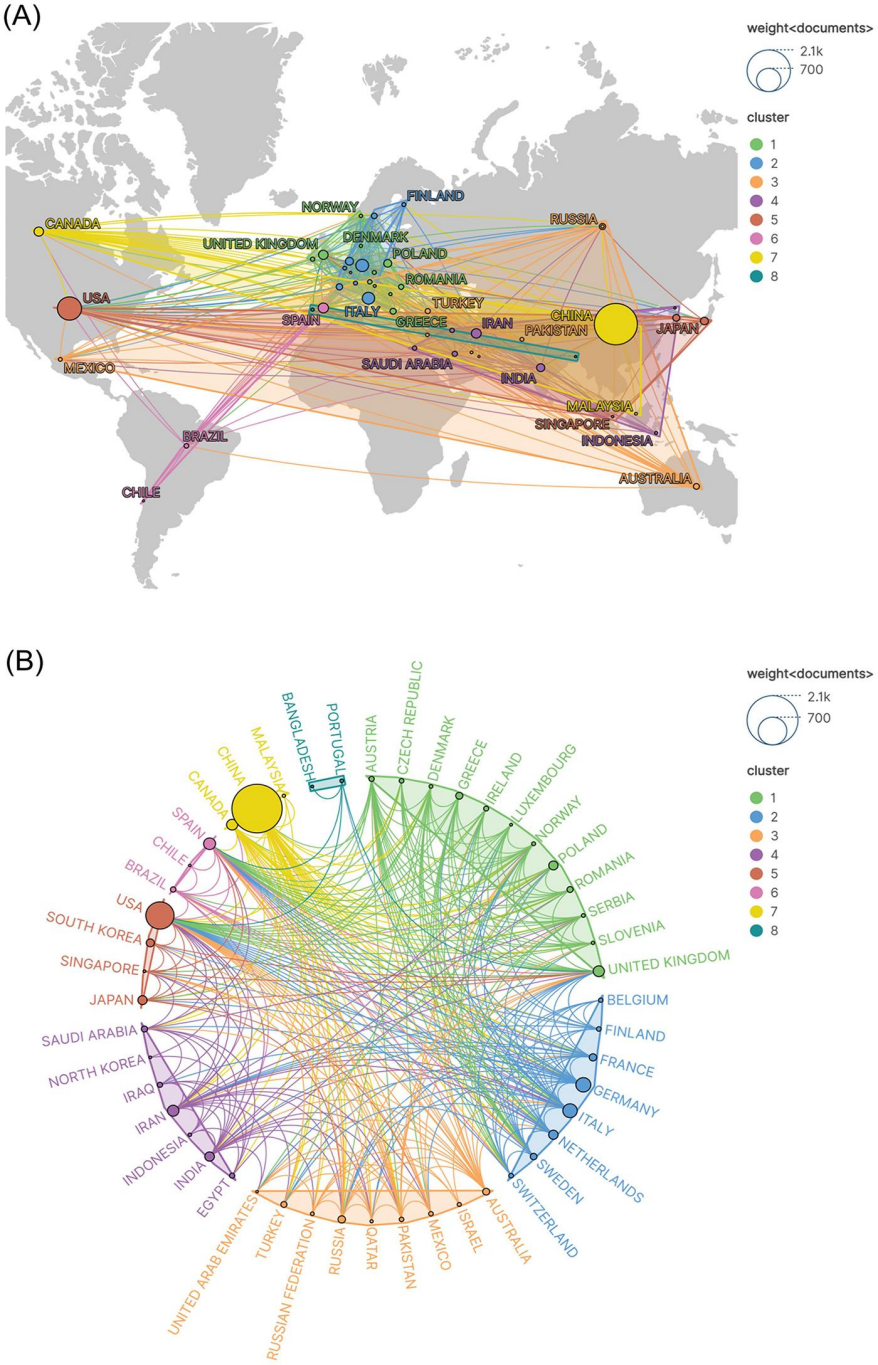
National and regional distribution and collaboration networks in microRNA and atherosclerosis research. **(A)** Geographic visualization of international research collaborations on a world map. **(B)** Circular network visualization of country-level research collaborations.

Figure 3A illustrates the global distribution of research activities, indicating that a wide range of countries/regions have contributed. Current trends suggest substantial potential for further expansion and deepening of the research scope. Figure 3B presents a visualization of the global scientific collaboration network among countries/regions. In this network, node size is proportional to each country/region's publication output, node color represents distinct collaborative clusters, and connecting lines indicate the intensity of inter-country/regional collaboration. In the visualization, China emerges as the largest node, reflecting the highest publication volume globally, followed by the United States; together, they serve as the core contributors to scientific output. European nations, including Germany and the United Kingdom, alongside key contributors such as India and Canada, also constitute important research forces. Global research efforts are organized into eight primary collaborative clusters: the yellow cluster, centered on China, extends to Asia-Pacific and neighboring countries, including Canada and Malaysia; the red cluster, led by the United States, connects with Asia-Pacific nations, including South Korea and Japan; and the purple cluster, anchored by India, spans multiple countries across the Middle East and South Asia. Each cluster exhibits stable regional collaborative dynamics. In terms of collaboration intensity, the most densely connected nodes involve core countries—China, the United States, the United Kingdom, and Germany—forming pivotal hubs that facilitate international academic collaboration. Notably, collaborative ties among European countries are particularly robust, reflecting a high degree of regional scientific coordination.

In the institutional-level bibliometric analysis, Table 4 presents the top 15 research institutions ranked by number of publications, all located in China, underscoring China's dominant role in scientific output in this field. Table 5 lists the top 15 globally cited research institutions, ranked by total citations. Regarding global academic influence, institutions from the United States and Germany are particularly prominent. For instance, New York University (USA) has published only 28 papers but received 5,130 citations, averaging 183 citations per paper. Its research has become a benchmark in the field owing to its high innovativeness and recognition. Additionally, German institutions, including RWTH Aachen University, Goethe University Frankfurt, and LMU Munich, rank among the top globally cited institutions despite their relatively small publication numbers, reflecting their innovative and rigorous research. This clearly demonstrates the profound academic accumulation, solid research foundation, and leading international role of the United States and Germany in this field. Six Chinese institutions also appear among the top 15 globally cited institutions. This outcome not only confirms the notable improvement in the academic influence and growing international recognition of Chinese institutions, which is concomitant with high scientific output, but also reflects the overall research landscape that integrates regional strengths with diverse global contributions.

**Table 4:** Top 15 organizations in terms of number of publications.

Rank	Organization	Documents	Citations	Total link strength	Country
1	Capital Medical University	61	1,523	53	CHINA
2	Sun Yat-sen University	61	2,495	73	CHINA
3	Harbin Medical University	54	1,680	22	CHINA
4	Shanghai Jiao Tong University	54	2,315	56	CHINA
5	Huazhong University of Science and Technology	52	2,075	38	CHINA
6	University of South China	50	1,647	68	CHINA
7	Fudan University	47	2,126	50	CHINA
8	Nanjing Medical University	47	2,854	40	CHINA
9	Zhengzhou University	45	1,237	17	CHINA
10	Qingdao University	39	1,190	21	CHINA
11	Central South University	38	1,193	28	CHINA
12	Jilin University	37	1,257	25	CHINA
13	Southern Medical University	36	1,130	56	CHINA
14	Zhejiang University	36	1,142	29	CHINA
15	Shandong University	61	1,523	53	CHINA

**Table 5:** Top 15 organizations ranked by citation rate.

Rank	Organization	Citations	Documents	Total link strength	Country
1	New York University	5,130	28	38	USA
2	RWTH Aachen University	3,141	14	37	Germany
3	Nanjing Medical University	2,854	47	40	China
4	Goethe University Frankfurt	2,555	9	6	Germany
5	Maastricht University	2,503	6	17	Netherlands
6	Sun Yat-sen University	2,495	61	73	China
7	King's College London	2,379	14	27	United Kingdom
8	University of California, San Francisco (UCSF)	2,376	9	4	USA
9	Shanghai Jiao Tong University	2,315	54	56	China
10	LMU Munich	2,163	12	29	Germany
11	University of Rochester	2,160	5	3	USA
12	Fudan University	2,126	47	50	China
13	Huazhong University of Science and Technology	2,075	52	38	China
14	Yale University	1,907	22	27	USA
15	Harbin Medical University	1,680	54	22	China

**Figure 4:**
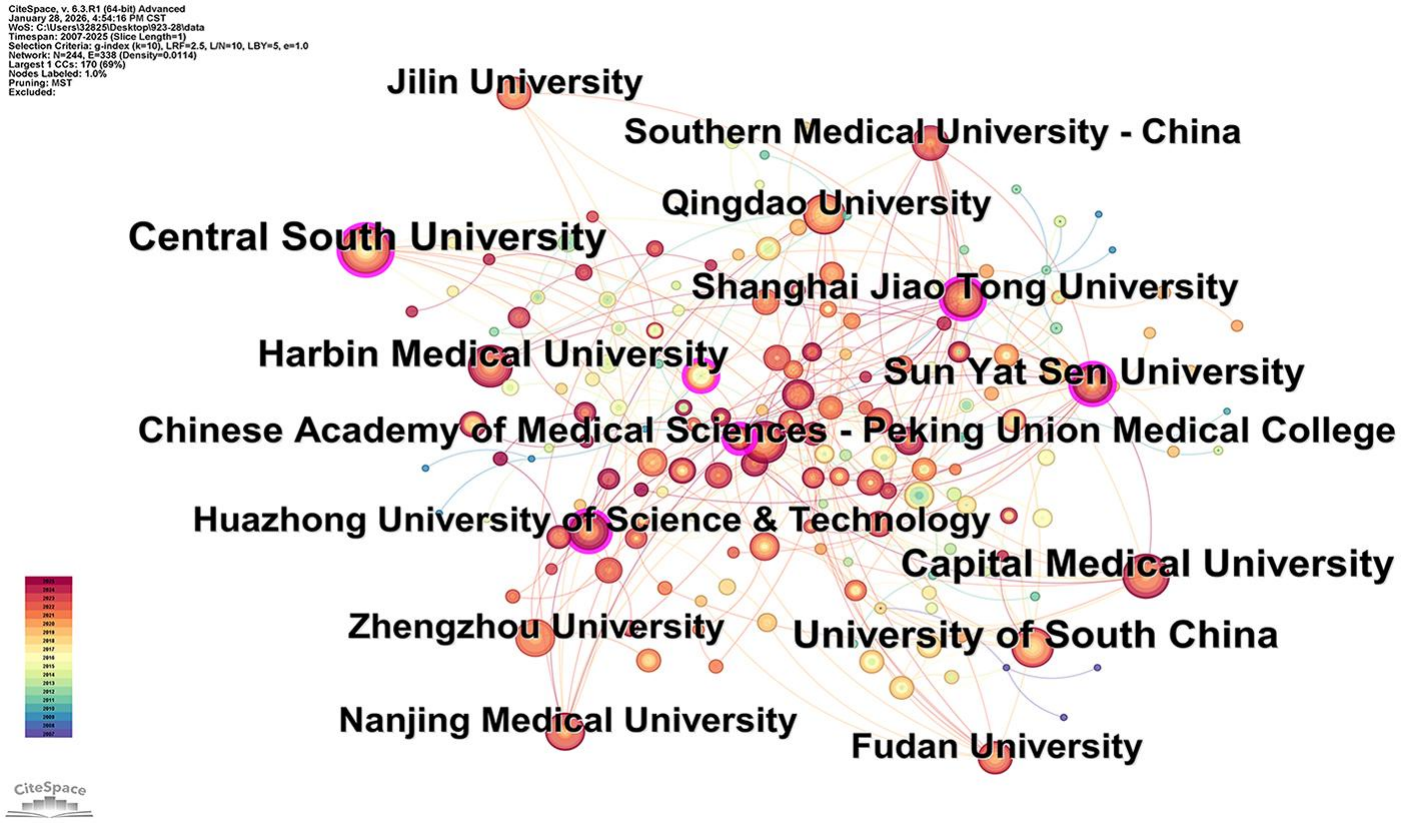
Institutional network co-occurrence map.

Figure 4 systematically depicts the distribution of research institutions within the field of miRNAs and AS. Each node represents an institution, with size positively correlated with publication output, visually reflecting institutional research activity. Node color gradients denote publication timelines, while connecting lines represent inter-institutional collaborations, collectively illustrating the overall structure of the research network.

### 3.3 Analysis of the geographic distribution of authors and co-authorship collaboration networks

A total of 13,223 authors have contributed to research on miRNAs in AS. Table 6 presents the top 15 authors ranked by the number of publications, with Christian Weber leading the list with 34 publications, followed by other prolific contributors such as Andreas Schober and Carlos Fernández-Hernando. In terms of collaborative engagement, scholars including Chao-Ke Tang, Min Zhang, and Xi-Long Zheng exhibit notably high total link strength, reflecting their extensive academic cooperation networks. International researchers such as Carlos Fernández-Hernando and Christian Weber also demonstrate strong collaborative capacity. Table 7 summarizes the top 15 authors ranked by total citation count, with Carlos Fernández-Hernando at the forefront with 4,252 citations, indicating the high recognition of his research output. Andreas Schober, Christian Weber, and Kathryn J. Moore are also regarded as benchmarks due to their influential citation records.

**Table 6:** Top 15 authors by number of publications.

Rank	Documents	Authors	Citations	Total link strength
1	34	Christian Weber	3,590	109
2	30	Andreas Schober	3,732	92
3	28	Carlos Fernández-Hernando	4,252	113
4	27	Chao-Ke Tang	973	265
5	24	Min Zhang	991	216
6	23	Xi-Long Zheng	839	238
7	21	Yu Wang	195	12
8	20	Jing Zhang	549	28
9	20	Lei Zhang	667	18
10	19	Noemi Rotllan	1,245	77
11	19	Seyedeh Maryam Hosseinikhah	24	11
12	19	Yajaira Suárez	2,709	90
13	17	Ping-Ping He	779	182
14	17	Lars Maegdefessel	1,500	35
15	17	Katey J. Rayner	2,686	54

**Table 7:** Top 15 authors ranked by citation rate.

Rank	Citations	Authors	Documents	Total link strength
1	4,252	Carlos Fernández-Hernando	28	113
2	3,732	Andreas Schober	30	92
3	3,665	Kathryn J Moore	15	45
4	3,590	Christian Weber	34	109
5	2,971	Kasey C Vickers	8	2
6	2,872	Stefanie Dimmeler	16	21
7	2,854	Edward A Fisher	9	33
8	2,709	Yajaira Suárez	19	90
9	2,686	Katey J Rayner	17	54
10	2,314	Andreas M Zeiher	5	9
11	2,131	Leigh Goedeke	16	69
12	2,059	Kiril Bidzhekov	8	31
13	2,043	Yuanyuan Wei	16	56
14	1,886	Manuel Mayr	13	34
15	1,765	Maliheh Nazari-Jahantigh	15	61

**Figure 5:**
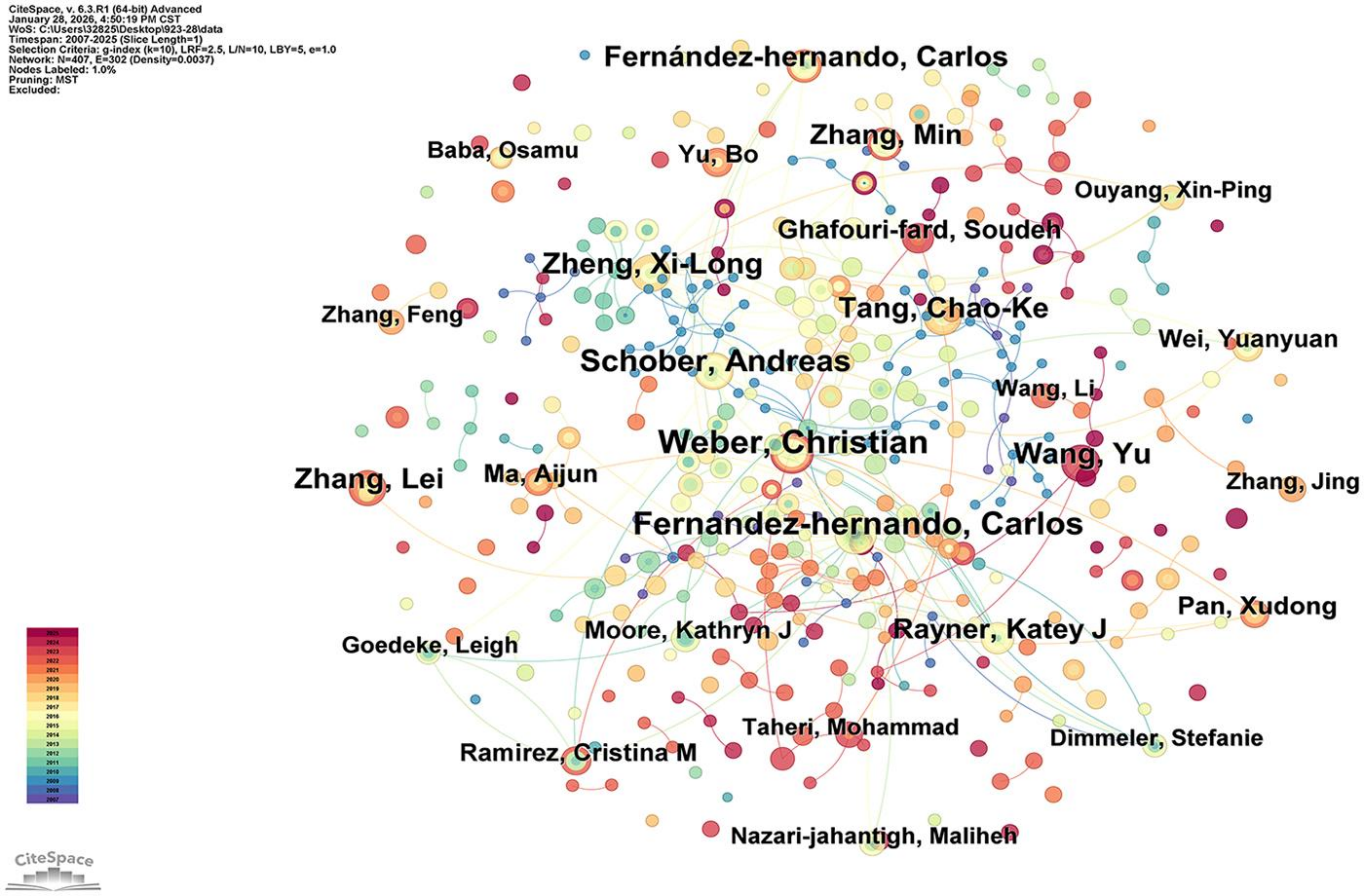
Author co-occurrence network view.

Figure 5 displays the author co-occurrence network, in which node size is proportional to each author's publication count. Christian Weber, a German cardiovascular researcher, initiated studies on miRNAs in the context of AS as early as 2009. His early work focused on the paracrine signaling functions of endothelial cell (EC) apoptotic vesicles in AS, leading to the identification of the “endothelial apoptotic vesicle–miRNA–G protein-coupled receptor” axis and validating the therapeutic potential of miR-126 (22). In subsequent studies, Weber further found that miR-145 can target and regulate the expression of Junctional adhesion molecule-A (JAM-A) to alleviate AS, while miR-155 exerts its inhibitory effect on AS progression by targeting macrophages (23, 24). Notably, various miRNAs form multilayered molecular networks that regulate endothelial function, VSMC phenotypes, macrophage-mediated inflammation, and lipid metabolism (25, 26). Targeting these miRNAs not only offers promising therapeutic strategies for AS but also provides valuable insights into the pathogenesis of related vascular disorders, including hypertension and restenosis (27, 28).

Carlos Fernández-Hernando, an internationally recognized medical scientist, specializes in CVDs, miRNA regulation, and lipid metabolism. In lipid metabolism and AS research, he achieved significant breakthroughs by systematically elucidating the core regulatory mechanisms of miR-33 in reverse cholesterol transport and fatty acid oxidation (12, 29). Through detailed analyses of miR-33 interactions with its key target genes, he and his collaborators developed an anti-miR-33 antisense oligonucleotide therapy. This therapy showed remarkable efficacy in both murine and non-human primate models, significantly increasing high-density lipoprotein cholesterol (HDL-C) levels, reducing very low-density lipoprotein (VLDL) concentrations, and decreasing atherosclerotic plaque burden. These findings have opened new avenues for the treatment of metabolic disorders (e.g., hyperlipidemia and metabolic syndrome) and provided an essential theoretical and experimental foundation for the clinical translation of RNA-targeted therapies (30, 31). It should be emphasized that long-term inhibition of miR-33 carries the risk of inducing hepatic steatosis, highlighting the urgent need to develop more precise regulatory strategies to balance therapeutic efficacy and safety (11).

Table 8 lists the top 15 most-cited authors, each with more than 170 citations. Their foundational work, though not always directly focused on the miRNA-AS axis, has been instrumental in shaping this interdisciplinary field. For instance, Peter Libby, a leading cardiovascular scientist at Harvard Medical School, established the central role of inflammation in AS. By elucidating the pivotal functions of immune cells and inflammatory mediators in vascular pathology (32–34), his work provided the essential disease context in which the regulatory roles of molecules like miRNAs would later be explored. Similarly, David P. Bartel, a pioneer in gene expression regulation, laid the methodological and theoretical groundwork for miRNA biology (35, 36). His work established the essential methodologies and techniques necessary for investigating the functions of miRNAs in a wide range of diseases, including CVDs. Therefore, although Libby and Bartel were not direct investigators in the miRNA-AS interdisciplinary field, their pioneering work in cardiovascular biology and miRNA regulation collectively formed the indispensable foundation upon which this cross-disciplinary area could develop.

**Table 8:** Top 15 authors of cited papers.

Rank	Citations	Total link strength	Cited Authors
1	622	4,196	Libby P
2	475	3,321	Bartel DP
3	394	4,887	Rayner KJ
4	301	1,902	Zhang Y
5	220	1,409	Wang Y
6	213	1,524	Feinberg MW
7	210	1,677	Hansson GK
8	209	1,948	Moore KJ
9	205	1,571	Tabas I
10	196	1,006	Livak KJ
11	187	1,996	Fichtlscherer S
12	182	1,822	Schober A
13	178	1,014	Chistiakov DA
14	176	1,699	Raitoharju E
15	175	1,039	Zhang L

In contrast, the research that bridges these two fields and systematically elucidates the specific roles of miRNAs in AS has been primarily advanced by scholars specializing in cross-disciplinary investigation, such as Katey J. Rayner. Katey J. Rayner, a leading Canadian researcher in cardiovascular medicine and miRNA biology, investigates the molecular mechanisms underlying AS and metabolic disorders, as well as the therapeutic potential of miRNAs. Her studies on miR-33 demonstrated its role in modulating AS pathogenesis through the regulation of cholesterol transporters and macrophage function (37–39).

### 3.4 Analysis of journals and cited journals

Table 9 indicates that the top 15 most productive journals published 727 articles, representing 20.90% of the total output, thereby underscoring their pivotal role in academic communication and research dissemination. Table 10 presents the top 15 journals ranked by h-index, providing a comprehensive overview of their bibliometric performance.

**Table 9:** Top 15 journals in terms of number of publications.

Source	NP	h_index	g_index	m_index	TC	PY_start	IF(2024)	Category Quartile
International Journal of Molecular Sciences	107	16	38	1.143	1,483	2013	4.9	Q1
Atherosclerosis	82	31	54	1.938	2,983	2011	5.7	Q1
Circulation Research	58	36	58	1.8	7,560	2007	16.2	Q1
Arteriosclerosis, Thrombosis, and Vascular Biology	56	40	56	2.353	5,342	2010	7.4	Q1
Frontiers in Cardiovascular Medicine	56	12	19	1.2	424	2017	2.9	Q2
Scientific Reports	52	20	38	1.538	1,516	2014	3.9	Q1
Biochemical and Biophysical Research Communications	44	28	44	1.647	2,368	2010	2.2	Q3
PLOS ONE	44	25	44	1.389	2,188	2009	2.6	Q2
Molecular Medicine Reports	39	21	33	1.4	1,161	2012	3.5	Q2
Cardiovascular Research	38	20	38	1.053	2,333	2008	13.3	Q1
Experimental and Therapeutic Medicine	32	14	19	1.4	445	2017	2.3	Q3
Life Sciences	31	15	24	0.938	609	2011	5.1	Q1
Journal of Cellular and Molecular Medicine	30	18	27	1.5	736	2015	4.2	Q2
Journal of Cardiovascular Pharmacology	30	14	18	0.875	407	2011	2.2	Q2
Journal of Cellular Physiology	28	17	28	1.063	1,006	2011	4.0	Q1

**Table 10:** Top 15 journals ranked by h-index.

Source	h_index	g_index	m_index	TC	NP	PY_start	IF(2024)	Category Quartile
Arteriosclerosis, Thrombosis, and Vascular Biology	40	56	2.353	5,342	56	2010	7.4	Q1
Circulation Research	36	58	1.8	7,560	58	2007	16.2	Q1
Atherosclerosis	31	54	1.938	2,983	82	2011	5.7	Q1
Cardiovascular Research	28	44	1.647	2,368	44	2010	13.3	Q1
Biochemical and Biophysical Research Communications	25	44	1.389	2,188	44	2009	2.2	Q3
PLOS ONE	21	33	1.4	1,161	39	2012	2.6	Q2
Molecular Medicine Reports	20	38	1.053	2,333	38	2008	3.5	Q2
Cellular Physiology and Biochemistry	20	23	1.667	1,064	23	2015	2.0	Q3
Scientific Reports	20	38	1.538	1,516	52	2014	3.9	Q1
International Journal of Molecular Medicine	18	26	1.385	828	26	2014	5.8	Q1
Journal of Cellular and Molecular Medicine	18	27	1.5	736	30	2015	4.2	Q2
European Review for Medical and Pharmacological Sciences	17	27	1.889	751	27	2018	NA	NA
Journal of Cellular Physiology	17	28	1.063	1,006	28	2011	4.0	Q1
Molecular and Cellular Biochemistry	17	24	1.214	590	27	2013	3.7	Q2
Biomedicine & Pharmacotherapy	16	19	1.455	831	19	2016	7.5	Q1

The primary research areas of these top journals are concentrated in cardiovascular and molecular sciences. Led by high-impact publications such as *Circulation Research* (IF = 16.2), the substantial proportion of Q1 journals underscores the cutting-edge nature of research in this domain. This distribution indicates that these journals collectively foster a broad spectrum of research spanning from molecular mechanisms to clinical applications.

**Figure 6:**
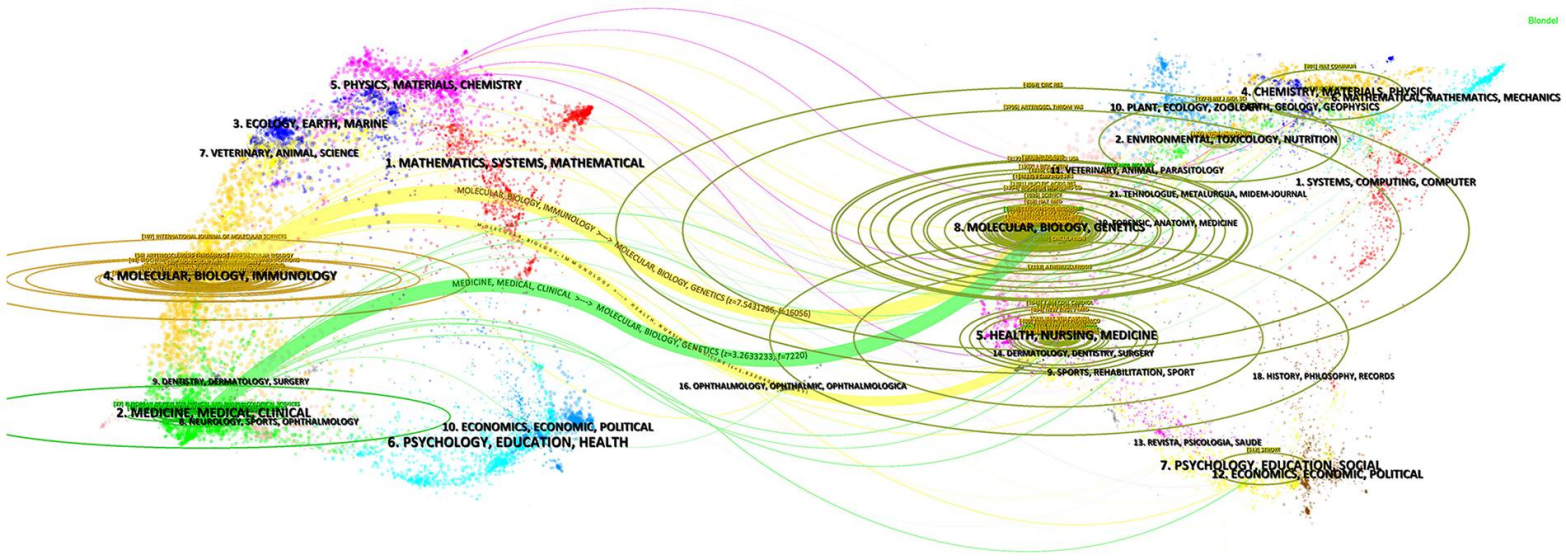
Dual-map overlay showing citation trajectories between citing and cited journals.

Figure 6 presents a dual-overlay map of journals, providing an intuitive and comprehensive visualization of inter-journal relationships. In this visualization, labels denote the core thematic areas of various journals, serving as reference points for understanding their research domains. The citation pathways vividly depict the interconnections among journals, symbolizing the knowledge linkages that integrate academic achievements across diverse publications. The analysis reveals three principal citation pathways: journals focused on “MOLECULAR, BIOLOGY, GENETICS” occupy a central position in the academic citation network, with their work frequently cited by journals addressing “MOLECULAR, BIOLOGY, IMMUNOLOGY” and “MEDICINE, MEDICAL, CLINICAL” themes; journals emphasizing “HEALTH, NURSING, MEDICINE” are also frequently cited by those concentrating on “MOLECULAR, BIOLOGY, IMMUNOLOGY.” This phenomenon reflects not only the broad influence and foundational role of research in “MOLECULAR, BIOLOGY, GENETICS,” but also the strong interconnections and mutual reinforcement of interdisciplinary studies, providing valuable insights for identifying emerging trends and future research directions.

Table 11 summarizes the characteristics of highly cited journals, with *Circulation Research* (2,337 citations) ranking first, underscoring the central position of cardiovascular research. The cited journals are predominantly clustered within the Q1 quartile, encompassing top-tier publications such as *Nature* (IF = 48.5) and *Cell* (IF = 42.5), reflecting researchers' preference for high-impact academic outputs.

**Table 11:** Top 15 most cited journals.

Citations	Total link strength	Cited Journals	IF(2024)	Category Quartile
4,507	246,351	Circulation Research	16.2	Q1
3,798	215,880	Arteriosclerosis, Thrombosis, and Vascular Biology	7.4	Q1
3,057	170,603	Circulation	38.6	Q1
2,225	129,212	PLOS ONE	2.6	Q2
2,172	125,479	Proceedings of the National Academy of Sciences of the United States of America	9.1	Q1
2,172	133,260	Nature	48.5	Q1
2,166	119,352	Atherosclerosis	5.7	Q1
1,940	116,118	The Journal of Biological Chemistry	3.9	Q2
1,816	96,901	Cell	42.5	Q1
1,629	103,338	The Journal of Clinical Investigation	13.6	Q1
1,622	88,643	Cardiovascular Research	13.3	Q1
1,381	76,238	Nucleic Acids Research	13.1	Q1
1,274	76,379	International Journal of Molecular Sciences	4.9	Q1
1,255	72,151	Biochemical and Biophysical Research Communications	2.2	Q3
1,216	73,176	Scientific Reports	3.9	Q1

### 3.5 Analysis of the most frequently cited references (based on local citation counts)

Table 12 presents the 15 most frequently cited references among the 3,478 original research articles included in this study. Local citation counts were defined as the number of times each reference was cited within the set of included original research articles. Although only original research articles were included as source documents, the types of references they cited were not restricted; thus, review articles may also appear among the most frequently cited references.

**Table 12:** Top 15 most frequently cited references in the included original research articles.

Rank	Title	First author	Journal	Citations	Year	IF(2024)	Category Quartile	Publication type
1	MicroRNAs: Genomics, Biogenesis, Mechanism, and Function	BARTEL DP	Cell	234	2004	42.5	Q1	Review
2	MicroRNA Regulation of Atherosclerosis	FEINBERG MW	Circulation Research	205	2016	16.2	Q1	Review
3	MicroRNAs: target recognition and regulatory functions	BARTEL DP	Cell	198	2009	42.5	Q1	Review
4	Analysis of relative gene expression data using real-time quantitative PCR and the 2(-Delta Delta C(T)) Method	LIVAK KJ	Methods	196	2001	4.3	Q1	Article
5	Circulating MicroRNAs in Patients With Coronary Artery Disease	FICHTLSCHERER S	Circulation Research	145	2010	16.2	Q1	Article
6	MiR-33 Contributes to the Regulation of Cholesterol Homeostasis	RAYNER KJ	Science	139	2010	45.8	Q1	Article
7	miR-21, miR-210, miR-34a, and miR-146a/b are up-regulated in human atherosclerotic plaques in the Tampere Vascular Study	RAITOHARJU E	Atherosclerosis	135	2011	5.7	Q1	Article
8	Endothelial Cell Dysfunction and the Pathobiology of Atherosclerosis	GIMBRONE MA	Circulation Research	130	2016	16.2	Q1	Review
9	MicroRNA expression signature and antisense-mediated depletion reveal an essential role of MicroRNA in vascular neointimal lesion formation	JI RR	Circulation Research	129	2007	16.2	Q1	Article
10	MicroRNA-155 promotes atherosclerosis by repressing Bcl6 in macrophages	NAZARI-JAHANTIGH M	Journal of Clinical Investigation	125	2012	13.6	Q1	Article
11	miR-145 and miR-143 regulate smooth muscle cell fate and plasticity	CORDES KR	Nature	120	2011	48.5	Q1	Article
12	Antagonism of miR-33 in mice promotes reverse cholesterol transport and regression of atherosclerosis	RAYNER KJ	Journal of Clinical Investigation	120	2009	13.6	Q1	Article
13	Progress and challenges in translating the biology of atherosclerosis	LIBBY P	Nature	116	2011	48.5	Q1	Article
14	MicroRNA-126-5p promotes endothelial proliferation and limits atherosclerosis by suppressing Dlk1	SCHOBER A	Nature Medicine	110	2014	50.0	Q1	Article
15	MicroRNA-33 and the SREBP Host Genes Cooperate to Control Cholesterol Homeostasis	NAJAFI-SHOUSHTARI SH	Nature	109	2010	48.5	Q1	Article

Notably, two highly cited reviews by Bartel published in *Cell* in 2004 and 2009 (234 and 198 local citations, respectively), provide comprehensive insights into miRNA biogenesis, target recognition, and regulatory functions, serving as foundational works for miRNA-related research (36, 40). Another frequently cited review is by Feinberg MW et al., published in *Circulation Research* in 2016 (205 local citations), which discusses the functional roles of miRNAs in AS and their potential implications for disease diagnosis and therapy (41).

In addition to these influential reviews, several key original research studies have contributed to the field in terms of technical methodology, clinical relevance, and molecular mechanisms. Livak KJ et al., published in *Methods* in 2001 (196 local citations), established the standardized 2−*ΔΔ*Ct method for quantitative PCR data analysis, providing a reliable technical foundation for miRNA expression studies in AS (42). Fichtlscherer S et al., published in *Circulation Research* in 2010 (145 local citations), characterized circulating miRNA profiles in patients with coronary artery disease—the primary clinical manifestation of AS—highlighting the relationship between miRNA expression and clinical phenotypes (43). Rayner KJ et al., published in *Science* in 2010 (139 local citations), demonstrated that miR-33 regulates cholesterol homeostasis by inhibiting ABCA1 and ABCG1 expression, thereby reducing cholesterol efflux and revealing a key pathway through which miRNAs contribute to lipid metabolic disorders in AS pathogenesis (12).

Overall, these highly cited references were predominantly published in top-tier journals, including *Cell*, *Circulation Research*, and *Science*, reflecting their high academic influence and recognition in the field.

**Figure 7:**
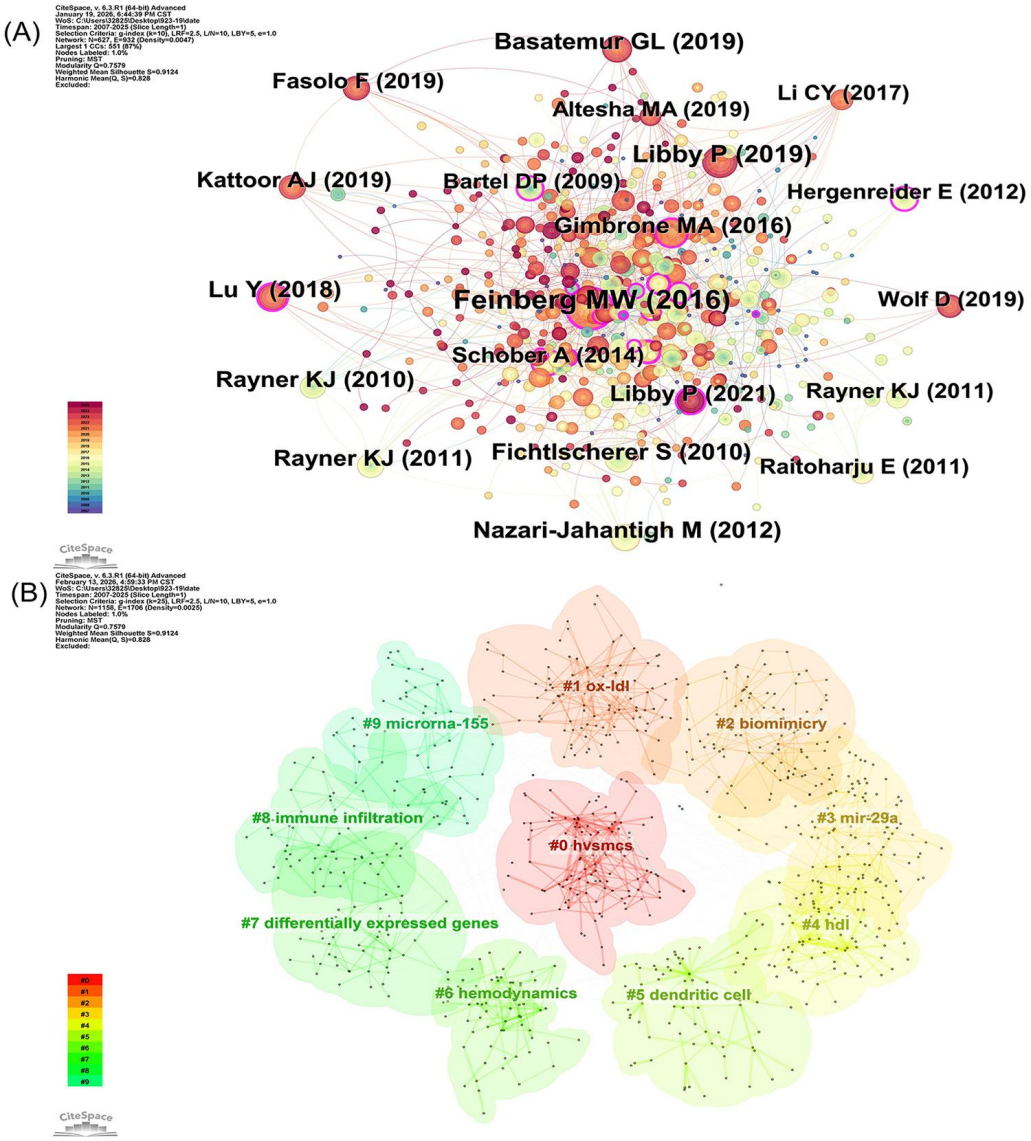
Analysis of references in microRNAs and atherosclerosis research. **(A)** Network co-occurrence graph of cited literature. **(B)** Cluster analysis graph of cited literature.

Figure 7A presents a co-citation network map, offering a unique perspective for understanding the interconnections among studies and the intellectual trajectory of academic development. Each node represents an academic paper, with node size proportional to citation frequency—larger nodes indicating greater academic influence. Node colors indicate publication years, allowing for an intuitive visualization of the temporal distribution of research. This visualization not only facilitates the identification of developmental trends but also elucidates the connections between different periods, highlighting the continuity of academic thought.

Figure 7B shows the cluster analysis of co-cited references in this field. The identified clusters represent distinct research themes: “hVSMCs” corresponds to VSMC function; “ox-LDL” and “hdl” are associated with lipid metabolism; “biomimicry” relates to biomimetic strategies for cardiovascular research; “mir-29a” and “microrna-155” represent specific microRNA regulatory studies; “dendritic cell” and “immune infiltration” reflect immune and inflammatory responses; “hemodynamics” corresponds to hemodynamic changes; and “differentially expressed genes” indicates gene expression regulation in cardiovascular diseases. These clusters collectively illustrate the major research directions in this field and reflect the multiple biological processes involved in the pathogenesis of AS.

### 3.6 Keyword analysis

This study analyzed 3,478 publications and extracted 5,419 authors' keywords, which cover the full spectrum of research topics in this domain. Table 13 presents the top 15 most frequently occurring keywords in this field, analyzed using VOSviewer. Among them, “atherosclerosis” appeared most frequently (1,551 occurrences; total link strength = 2,994), highlighting its key role as a core research subject in the field. “miRNA” ranked second (1,010 occurrences; total link strength = 2,467). Other high-frequency keywords include “inflammation,” “cardiovascular disease,” and “biomarker,” as well as cell-specific terms such as “endothelial cells” and “macrophages,” which collectively reveal the core cellular and molecular mechanisms driving disease progression. Furthermore, the emergence of emerging keywords such as “exosome,” “lncRNA,” and “circRNA” further reflects a growing research focus on novel intercellular communication pathways and complex non-coding RNA regulatory networks.

**Table 13:** Top 15 most frequently occurring keywords.

Rank	Occurrences	Total link strength	Keywords
1	1,551	2,994	atherosclerosis
2	1,010	2,467	miRNA
3	288	746	inflammation
4	221	590	cardiovascular disease
5	214	563	biomarker
6	164	432	endothelial cells
7	164	436	macrophages
8	163	346	vascular smooth muscle cells
9	134	221	ox-LDL
10	129	353	exosome
11	127	272	coronary artery disease
12	104	290	lncRNA
13	108	265	circRNA
14	106	257	apoptosis
15	101	235	proliferation

**Figure 8:**
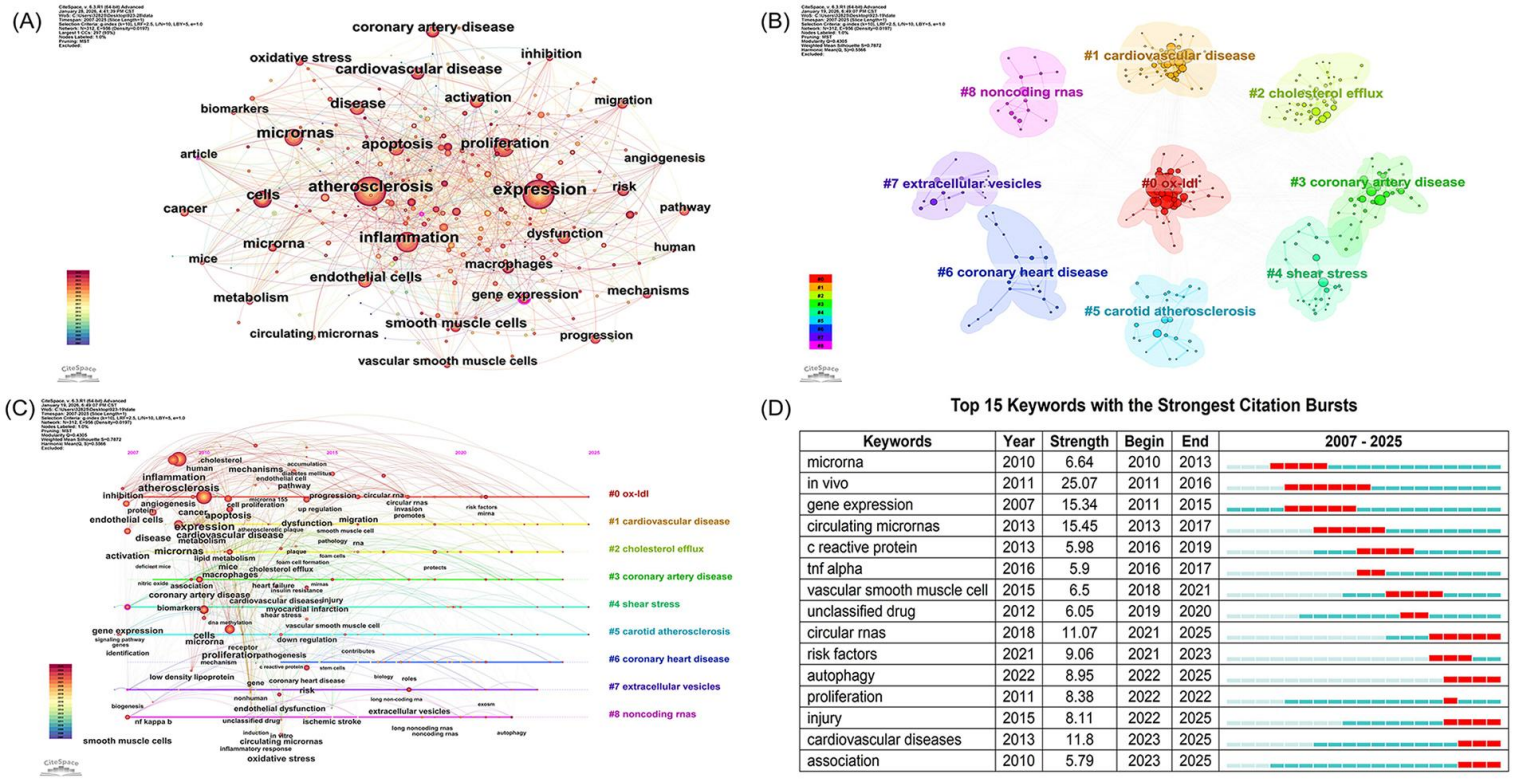
Keyword analysis in microRNAs and atherosclerosis research. **(A)** Keyword co-occurrence network. **(B)** Keyword cluster analysis. **(C)** Keyword timezone view. **(D)** Top 15 keywords with the strongest citation bursts.

Figure 8A presents the keyword co-occurrence network map, which intuitively illustrates that research in this field centers on AS, comprehensively covering disease mechanisms, molecular regulation, cellular dysfunction, and translational applications—with all research directions exhibiting robust interconnections. AS represents the core node, with related conditions like coronary artery disease and broader CVDs forming key interconnected nodes. Keywords such as inflammation, oxidative stress, apoptosis, proliferation, and metabolism indicate that elucidating the pathological mechanisms of AS constitutes a major research focus. Furthermore, keywords such as miRNAs, expression, endothelial cells, and macrophage indicate that research extends to molecular and cellular levels, with a particular emphasis on the regulatory role of miRNAs in AS development and progression. Keywords such as biomarkers, inhibition, and mechanisms further suggest that the identification of diagnostic biomarkers, therapeutic targets, and mechanistic exploration remain major research hotspots.

Figure 8B shows the keyword cluster network visualization. Nine major keyword clusters were identified, including #0 ox-LDL, #1 cardiovascular disease, #2 cholesterol efflux, #3 coronary artery disease, #4 shear stress, #5 carotid atherosclerosis, #6 coronary heart disease, #7 extracellular vesicles, and #8 noncoding RNAs. Research in this field spans multiple levels, from macro-scale CVDs and vascular pathologies to micro-scale molecular mechanisms, encompassing lipid metabolism disorders, vascular biomechanics, intercellular communication, and non-coding RNA regulation. This reveals a comprehensive and interdisciplinary research landscape centered on the pathogenesis and progression of atherosclerotic CVDs.

Figure 8C presents a keyword timeline visualization, delineating the evolution of miRNA-centric research in AS from 2007 to 2025. In the initial phase (2007–2010), studies primarily focused on the role of miRNAs in regulating fundamental pathological mechanisms of AS, such as inflammation and endothelial dysfunction. During the subsequent period (2010–2018), the research emphasis expanded to include miRNA-mediated regulation of lipid metabolism and their investigation as circulating biomarkers for translational potential. In the recent phase (2018–2025), the field has demonstrated a trend toward deep interdisciplinary integration, wherein miRNA research converges with novel delivery systems such as extracellular vesicles, driving the field toward a more profound mechanistic understanding.

Figure 8D presents the top 15 keywords with the strongest citation bursts in atherosclerotic cardiovascular disease research from 2007 to 2025, capturing the dynamic shifts in research focus over this period. In the early foundational phase (2007–2015), *in vivo* (burst strength = 25.07) and *gene expression* (burst strength = 15.34) were the most prominent, reflecting the reliance on *in vivo* experiments and gene expression profiling in early mechanistic studies. Notably, *microrna* emerged as an early burst keyword (2010–2013), confirming its sustained core role in the field. During the mid-phase (2015–2020), *circulating micrornas* (burst strength = 15.45) and *vascular smooth muscle cell* (burst strength = 6.5) became focal points, indicating a shift toward translational research in circulating biomarkers and vascular cell dysfunction. In the most recent phase (2020–2025), *circular rnas* (burst strength = 11.07), *autophagy* (burst strength = 8.95), and *cardiovascular diseases* (burst strength = 11.8) represent the latest frontiers, underscoring a growing emphasis on novel non-coding RNA regulators, cellular stress responses, and clinically relevant disease outcomes.

## 4 Discussion

### 4.1 Basic information about this study

In this study, a comprehensive bibliometric and visualization analysis of miRNA research in the AS field was performed using five specialized tools, including R-Studio, CiteSpace, VOSviewer, WPS, and SciMAgo Graphica. Through a rigorous literature screening strategy, a total of 3,478 highly relevant publications were identified. These tools enabled multidimensional data mining, integration, and visualization, thereby elucidating key research themes, developmental trajectories, and emerging trends in miRNA-related AS research. The visualization results provide robust data support and serve as an important reference for subsequent in-depth investigations and academic discussions. Furthermore, by examining annual publication trends, collaboration networks, and co-citation patterns, this study systematically delineates recent advances in miRNA research focused on AS.

In 2005, the introduction of 454 sequencing technology, a representative second-generation sequencing platform, marked the advent of the high-throughput sequencing era (44). The widespread adoption of sequencing platforms such as Solexa/Illumina in 2008 enabled large-scale detection and systematic analysis of miRNA expression profiles, establishing the technical foundation for subsequent studies (45). Between 2007 and 2012, research on miRNAs in AS was still at an early exploratory stage. During this period, the publication of the CRISPR-Cas9 gene-editing technique in *Science* (2012) represented a major technological milestone (46). In 2013, the successful application of CRISPR-Cas9 in mammalian cell models provided a precise tool for elucidating the functional mechanisms of miRNAs (47). Since 2013, the field has entered a phase of rapid expansion, driven by breakthroughs in high-throughput sequencing and gene-editing technologies, with the annual number of publications peaking at 441 in 2021.

Subsequently, research in this domain has flourished, with its scope continuously broadening and deepening. Rather than focusing solely on the biological regulation of miRNAs in vascular EC, macrophages, and VSMC, researchers have increasingly explored their potential as novel diagnostic biomarkers to enable early and accurate disease detection through miRNA expression profiling. On the therapeutic front, miRNAs have emerged as potential targets, prompting extensive efforts to develop innovative miRNA-based interventions that overcome the limitations of conventional treatments. This bidirectional exploration—spanning basic mechanisms and clinical applications—has substantially enriched the field and accelerated the advancement of miRNA research in AS and related CVDs toward greater depth and breadth.

### 4.2 Research hotspots

#### 4.2.1 Atherosclerotic disease clusters

Through in-depth keyword co-occurrence and clustering analyses, several disease categories that have attracted substantial research attention in this field were revealed. Coronary heart disease (CHD), a highly prevalent cardiovascular disorder, has been extensively investigated owing to its complex pathophysiology and substantial global disease burden. Acute myocardial infarction (AMI), characterized by sudden onset and high mortality, remains a major focus of intensive clinical and mechanistic investigations. Research on cerebral ischemia has largely concentrated on neuroprotective and reperfusion strategies aimed at improving outcomes in patients with cerebral ischemic stroke (CIS). Abdominal aortic aneurysms (AAAs) continue to represent a prominent research hotspot due to their high risk of rupture, particularly in the context of imaging-based diagnosis and surgical management. In addition, the formation and progression of atherosclerotic plaques constitute a central focus for elucidating vascular pathology, involving key mechanisms such as dysregulated lipid metabolism, chronic inflammation, and associated molecular pathways. Collectively, these disease entities and research directions delineate the major research hotspots in the field, underscoring their significant clinical relevance and scientific importance.

#### 4.2.2 Research on disease mechanisms

The pathogenesis of atherosclerosis is primarily driven by the interplay between lipid accumulation and inflammatory responses within the arterial wall (48–50). Lipid deposition promotes the recruitment of immune cells, which release pro-inflammatory mediators, thereby exacerbating endothelial dysfunction and further lipid accumulation. This process establishes a self-perpetuating vicious cycle that accelerates disease progression. As atherosclerosis advances, plaques within the vessel wall progressively lose stability under sustained inflammatory stimulation and become increasingly vulnerable to rupture. Plaque rupture exposes subendothelial components, leading to rapid activation of the coagulation cascade and platelet aggregation, ultimately resulting in thrombus formation. These thrombi may embolize and occlude coronary or cerebral arteries, potentially triggering acute myocardial infarction or cerebral ischemic stroke and causing severe clinical consequences.

##### 4.2.2.1 Inflammation and immune regulation

Numerous miRNAs regulate inflammatory signaling through post-transcriptional targeting of specific genes, thereby modulating key pathways such as nuclear factor-*κ*B (NF-*κ*B) and mitogen-activated protein kinase (MAPK). These interactions alter cytokine and chemokine expression and influence the activation and recruitment of inflammatory cells. Such regulatory networks exhibit high specificity and complexity, with distinct miRNAs exerting stage-dependent effects across different phases of atherosclerosis development.

Among pro-inflammatory regulators, miR-155 potently enhances NF-*κ*B signaling by targeting and inhibiting B-cell lymphoma 6 (Bcl6) and suppressor of cytokine signaling 1 (SOCS1), thereby promoting the release of inflammatory mediators such as interleukin-6 (IL-6) and tumor necrosis factor-α (TNF-α) and contributing to AS progression (51, 52). In addition, miR-33 modulates the AMPK/SIRT1 axis, thereby amplifying vascular inflammation and exacerbating foam cell formation through repression of the cholesterol efflux transporters ABCA1 and ABCG1 (38).

Conversely, several miRNAs exert anti-inflammatory effects by precisely targeting key components of inflammatory signaling cascades, thereby conferring therapeutic potential for mitigating AS progression. For example, miR-146a negatively regulates tumor necrosis factor receptor-associated factor 6 (TRAF6) and interleukin-1 receptor-associated kinase 1 (IRAK1), attenuating downstream inflammatory signaling and reducing pro-inflammatory cytokine production (53, 54). MiR-223 suppresses macrophage pro-inflammatory activity and promotes polarization toward an anti-inflammatory M2 phenotype, thereby attenuating AS progression (55). Members of the let-7 family are abundantly expressed in vascular EC (56). They enhance endothelial nitric oxide synthase (eNOS) activity and inhibit NF-*κ*B pathway activation, thus reducing endothelial apoptosis (57). MiR-26b exerts atheroprotective effects by directly targeting AnnexinA2, which in turn suppresses inflammatory responses and promotes collagen degradation to attenuate atherosclerotic lesion formation (58). MiR-590-5p directly targets Krüppel-like factor 12 (KLF12) and inhibits its expression, reduces the accumulation of p300 in the nucleus, and ultimately suppresses the activation of the NF-*κ*B signaling pathway, thereby exerting anti-inflammatory and anti-atherosclerotic effects (59).

Additionally, miRNAs modulate immune cell function and thereby influence AS progression. For instance, miR-181a targets TGF-*β*-activated kinase 1-binding protein 2 (TAB2) and the NF-*κ*B essential modulator (NEMO), thereby attenuating NF-*κ*B activation and alleviating vascular inflammation (60).

Collectively, these findings highlight miRNAs as molecular “switches” in AS pathology, whose bidirectional regulatory roles underscore their considerable potential as targets for precision-based therapeutic intervention strategies.

##### 4.2.2.2 Regulation of lipid metabolism

It has been well established that miRNAs precisely regulate cholesterol uptake, synthesis, transport, and excretion by interacting with their target genes, thereby maintaining cholesterol homeostasis (61, 62). MiR-33 inhibits the expression of ABCA1, ABCG1, and Niemann-Pick type C1 protein (NPC1), thereby impeding cholesterol efflux and transport (63–65). In animal models, anti-miR-33 enhances HDL-C functionality and reduces plaque burden (66). MiR-122 suppresses the LKB1/AMPK signaling pathway by targeting sirtuin 1 (SIRT1), thereby affecting hepatic cholesterol synthesis and metabolism (67). MiR-103-5p promotes intracellular lipid accumulation by targeting PLSCR4 and PANK3 genes in goat models (68). MiR-144 directly binds to ABCA1 mRNA, reducing ABCA1 protein synthesis by destabilizing the transcript or inhibiting its translation, thereby modulating cholesterol metabolism (69). MiR-148a modulates lipid metabolism by regulating the expression of low-density lipoprotein receptor (LDLR) and ABCA1 (70). Experimental evidence indicates that inhibition of miR-148a effectively reduces atherogenic low-density lipoprotein cholesterol (LDL-C) while elevating anti-atherosclerotic HDL-C, offering a promising therapeutic approach for lipid metabolic disorders (71). MiR-7683-3p exerts a protective effect against AS by targeting HOXA1 and activating the PPAR*γ*-LXR*α*-ABCG1-mediated cholesterol efflux pathway, thereby regulating lipid metabolism in vascular smooth muscle cell-derived foam cells and enhancing atherosclerotic plaque stability (72). Moreover, miR-30c downregulates microsomal triglyceride transfer protein (MTP) mRNA and targets lysophosphatidylglycerol acyltransferase 1 (LPGAT1), thereby reducing plasma cholesterol levels and providing a potential therapeutic target for hyperlipidemia and AS (73).

##### 4.2.2.3 Regulation of cellular behavior

In vascular physiology and pathology, miRNAs play critical roles in regulating VSMC proliferation and migration through specific molecular targeting mechanisms. By interacting with their target mRNAs, miRNAs precisely modulate cell proliferation signaling pathways and the expression of migration-related proteins, thereby contributing to the development of AS and restenosis. MiR-21-3p has been identified as a key regulatory molecule that targets phosphatase and tensin homolog (PTEN), thereby activating downstream signaling cascades and enhancing VSMC migration and proliferation (74). Such aberrant cellular behaviors disrupt vascular homeostasis, exacerbate inflammatory responses, and ultimately accelerate the progression of AS. In atherosclerotic plaques, miR-155 expression is markedly upregulated, aggravating disease severity by targeting eNOS and promoting VSMC proliferation and migration (75). Notably, miR-29a promotes the proliferation and migration of human aortic smooth muscle cells (HASMCs) by targeting proliferating cell nuclear antigen (PCNA) and cell cycle protein D1 (cyclin D1), thereby accelerating atherosclerotic progression (76). Conversely, miR-125b targets serum response factor (SRF), inhibiting VSMC proliferation and migration and attenuating vascular neointima formation (77). MiR-15a-5p directly targets Sema3A to inhibit its expression, and its aberrant upregulation accelerates VSMC phenotypic switching, exacerbates macrophage infiltration and lipid accumulation in plaques, and is a key mediator of atherosclerotic plaque instability (78).

Moreover, miRNAs critically regulate EC function and structural integrity. MiR-199a-3p modulates autophagy by targeting the mechanistic target of rapamycin (mTOR) signaling pathway, thereby suppressing inflammatory and adhesion responses in vascular EC, maintaining endothelial homeostasis, and alleviating inflammatory injury (79). Conversely, miR-92a activates pro-inflammatory signaling by disrupting normal EC physiological functions, thereby contributing to atherosclerotic progression (80). Furthermore, miR-34a-5p specifically targets SIRT1 mRNA, reducing its expression and subsequently impairing nitric oxide synthesis and vasodilatory capacity, thereby exacerbating endothelial dysfunction and accelerating vascular injury (81). MiR-3529-3p promotes atherosclerotic progression by inducing ferritin heavy chain 1 (FTH1)-dependent ferroptosis in vascular EC (82).

Collectively, these findings highlight miRNAs as pivotal regulators of vascular cell phenotypes, orchestrating VSMC proliferation, migration, and endothelial dysfunction, which together drive plaque progression and vascular remodeling during atherosclerosis.

#### 4.2.3 Transformation and application

##### 4.2.3.1 Biomarker development

Emerging evidence supports the potential utility of disease-specific miRNA expression signatures as biomarkers in atherosclerosis. In nonhuman primate models of early- to mid-stage atherosclerosis, several miRNAs, including miR-5001 and miR-7975, have been identified as candidate biomarkers and therapeutic targets (83). The expression profiles of miRNAs in patients with early-onset coronary artery disease differ significantly from those in healthy individuals, as revealed by comparative analyses. Among patients matched for age and sex, significant downregulation of miR-145 in vascular EC and upregulation of miR-126 in vascular epithelial cells have been observed (84). These disease-specific miRNA alterations have been proposed as biomarkers for early diagnosis and disease stratification in coronary artery disease.

According to Barbalata et al., plasma levels of miR-142, miR-223, miR-155, and miR-92a have been shown to effectively predict postoperative cardiovascular risk in patients with peripheral arterial disease undergoing femoral artery bypass grafting (85). Notably, plasma miR-142 serves as an independent prognostic biomarker, allowing clinicians to more accurately evaluate patient prognosis and design personalized treatment strategies. Similarly, Wang et al. reported that plasma exosomal miR-30e and miR-92a are closely associated with AS, and their expression levels may serve as important indicators for disease onset and progression (86). In addition, various miRNAs, such as miR-155 and miR-21, are closely associated with inflammatory responses. Their expression levels increase in a dose-dependent manner with the severity of inflammation and are markedly upregulated in AS lesions (87–89). Monitoring the expression of these miRNAs enables effective assessment of atherosclerotic plaque stability and inflammatory status, thereby facilitating disease severity evaluation.

##### 4.2.3.2 Clinical therapeutic applications

With increasing insight into the pathological and regulatory roles of miRNAs in AS, therapeutic agents targeting pathogenic miRNAs have emerged as promising treatment strategies. Antisense oligonucleotide (ASO) therapies have been developed to antagonize miR-33 function, enhance reverse cholesterol transport, and reduce plasma cholesterol levels, thereby decelerating AS progression (90). Targeted delivery of miR-33 ASOs to atherosclerotic plaques using pH-low insertion peptides (pHLIPs) markedly enhances targeting efficiency and bioactivity, resulting in effective inhibition of miR-33–mediated atherogenic processes and attenuation of disease progression (66).

In another approach, selective multifunctional nanoparticles engineered to deliver anti-miR-712 specifically to inflamed aortic EC in murine models resulted in significant attenuation of vascular inflammation and inhibition of AS development (91). Other studies have identified that miR-10a restores mitochondrial respiration in atherosclerotic macrophages to increase chromatin accessibility and reinitiate their polarization toward an anti-inflammatory phenotype. A miR-10a-delivering nanoparticle targeting pro-inflammatory macrophages in atherosclerotic plaques was further developed, which effectively alleviated AS progression in murine models and thus provided a novel metabolically based epigenetic modulation strategy and targeted delivery tool for AS therapy (92).

These miRNA-targeted therapeutic strategies enable precise modulation of pathogenic miRNAs within diseased vascular tissues, offering enhanced specificity and reduced adverse effects due to minimized off-target interactions compared with conventional therapies. Collectively, these innovative approaches represent promising therapeutic avenues for AS and are expected to play a pivotal role in future CVDs management.

## 5 Limitations

(1) The analysis was conducted using three major databases (WoSCC, Scopus, and PubMed), potentially resulting in the omission of relevant studies from other sources. Incorporating additional databases, such as Embase, could reduce selection bias and broaden coverage in future research. (2) These databases predominantly index high-impact English-language journals, potentially excluding non-English publications. Development of bibliometric tools with multilingual capabilities could address this limitation in future investigations. (3) This study included only articles, excluding other publication types such as conference papers and book chapters, which may have overlooked potentially important findings. Integrating diverse literature types could provide a more comprehensive perspective in subsequent studies. (4) Reference analysis of PubMed-derived data was limited by software incompatibilities, potentially affecting the completeness of field mapping. (5) Despite systematic analysis of overall research characteristics and trends, subgroup analyses for key directions—such as miRNA-mediated regulation in ECs and VSMCs, miRNA roles in immune responses during atherogenesis, and miRNA regulation of lipid metabolism—were not performed. Consequently, detailed exploration of heterogeneity and specific developmental patterns within these subfields remains unaddressed. (6) A temporal lag exists in the dataset, with the most recent studies potentially absent. Inclusion of recent publications in future analyses could further refine the understanding of field development.

## 6 Summary

Through a comprehensive bibliometric analysis, this study demonstrates a steadily growing research interest in the role of miRNAs in AS, revealing distinct developmental trajectories and emerging research hotspots. Current evidence indicates that miRNAs are extensively involved in the pathophysiological processes of AS and play central roles in regulating inflammation, immune responses, lipid metabolism disorders, and vascular cell proliferation and migration.

Future research on miRNAs in the context of AS is expected to increasingly focus on translational and clinical applications. On one hand, quantifying specific miRNA expression levels in serum may contribute to the establishment of novel biomarker systems for early diagnosis, disease monitoring, and prognostic evaluation of AS. On the other hand, clinical therapies are anticipated to emphasize the development of innovative drug delivery materials and the construction of highly efficient, targeted delivery systems, enabling precise miRNA-based interventions at atherosclerotic lesion sites. Continued investigation into the associations between miRNAs and AS, alongside the elucidation of their underlying molecular mechanisms, will open new avenues for the prevention and treatment of AS-related diseases and foster significant progress in CVDs management.

## Data Availability

The original contributions presented in the study are included in the article/Supplementary Material, further inquiries can be directed to the corresponding author/s.
